# Supplementary material for: Routine sub-2.5 Å cryo-EM structure determination of GPCRs
Source: Nat Commun. 2021 Jul 15;12:4333. doi: 10.1038/s41467-021-24650-3 (PMC8282782; doi:10.1038/s41467-021-24650-3)
Supplement: Supplementary file 1 — Supplementary Information [file 41467_2021_24650_MOESM1_ESM.pdf]

Supplementary Information

for

Routine sub-2.5 Å cryo-EM structure determination of GPCRs

Radostin Danev*, Matthew Belousoff, Yi-Lynn Liang, Xin Zhang, Fabian Eisenstein, Denise Wootten, Patrick M. Sexton

*correspondence to: rado@m.u-tokyo.ac.jp

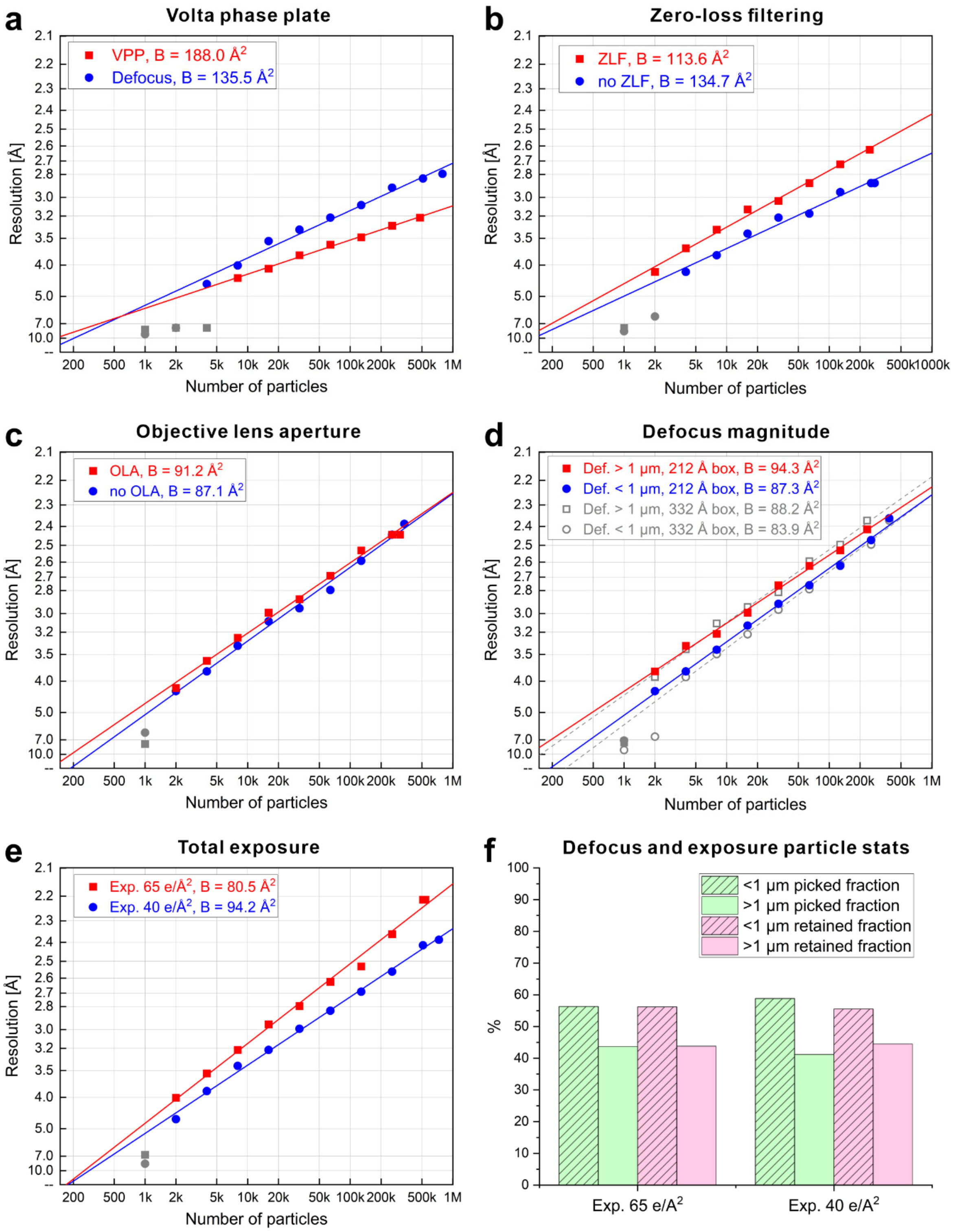

Supplementary Figure 1. B-factor plots for all particle subsets and particle retention statistics for the total exposure subsets.

a B-factor plots for the VPP/defocus subsets of the PAC1R dataset. **b** B-factor plots for the zero-loss energy filtering and no-energy-filtering subsets of the GLP-1R-TAS dataset. **c** B-factor plots for the objective lens aperture/no aperture subsets of the GLP-1R-GLP-1 dataset. **d** B-factor plots for the below and above 1 µm defocus subsets of the GLP-1R-GLP-1 dataset. The graph also contains B-factor plots for the defocus subsets reconstructed with a 332 Å box. **e** B-factor plots for the full and partial total exposure micrograph sets from the GLP-1R-GLP-1 dataset. **f** Defocus magnitude particle fractions as picked (green) and in the final refinement set (pink) for the full and partial total exposure sets from the GLP-1R-GLP-1 dataset.

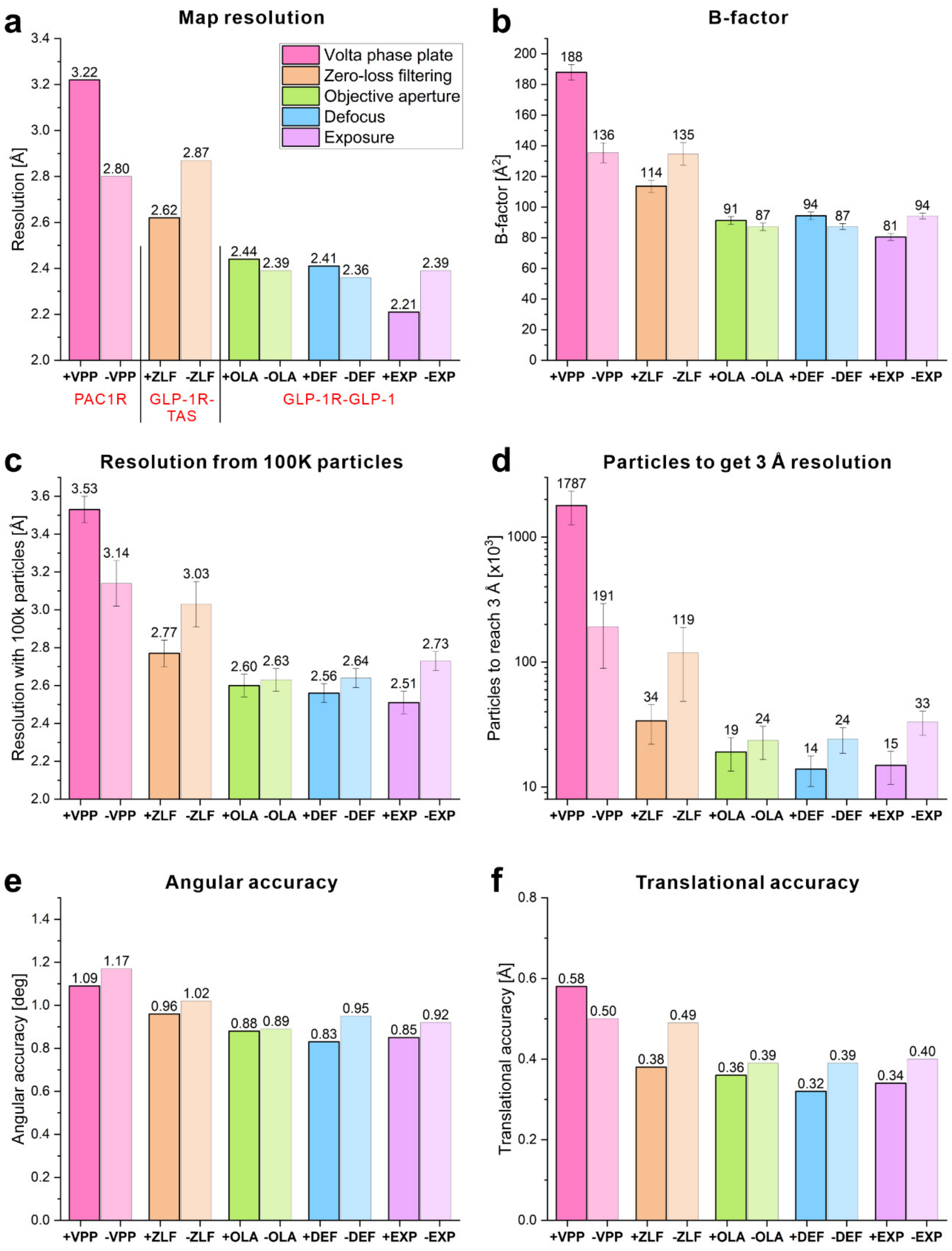

Supplementary Figure 2. Graphical summary of the performance test results.

a Resolution of the reconstructions from the particle subsets. The results between samples are not directly comparable (sample names are shown below the horizontal axis). **b** B-factor of each subset measured from the B-factor plots in Supplementary Figure 1. **c** Resolution from 100k particles in each subset, calculated from the linear fits of the B-factor plots in Supplementary Figure 1. **d** Number of particles in each subset required to reach 3 Å resolution, calculated from the linear fits of the B-factor plots in Supplementary Figure 1. **e** Angular alignment accuracy from the last 3D auto-refinement in Relion for each subset, as reported by the *rlnAccuracyRotations* value in the "run_model.star" file. Lower values indicate better accuracy. **f** Translational alignment accuracy from the last 3D auto-refinement in Relion for each subset, as reported by the *rlnAccuracyTranslationsAngst* value in the "run_model.star" file. Lower values indicate better accuracy. Error bars represent the standard error of each value estimated from the B-factor linear fits through $7 \leq n \leq 9$ independent 3D reconstructions from random particle subsets of varying size (Supplementary Figs. 1a-e).

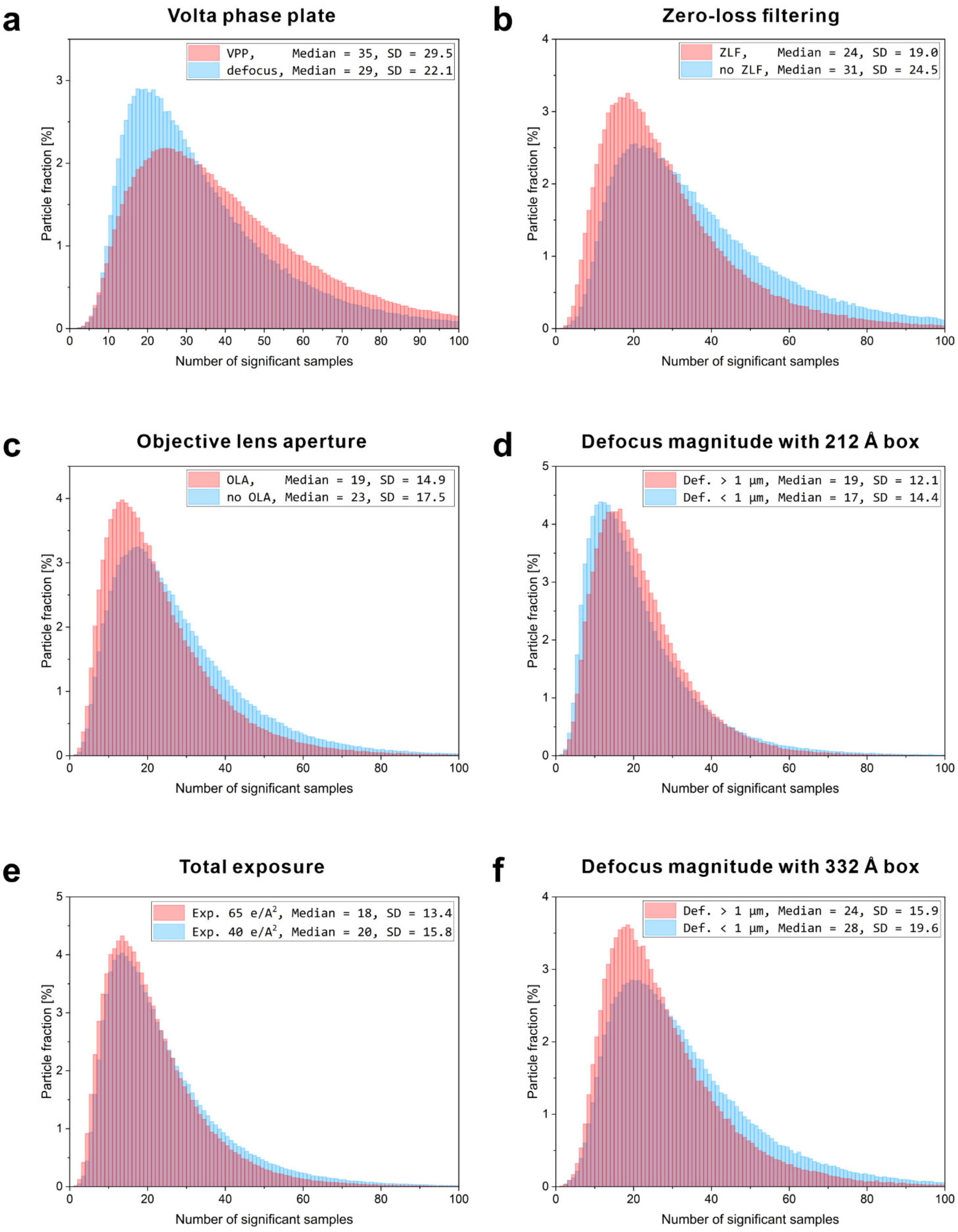

Supplementary Figure 3. Alignment statistics for the final particle sets.
a-f Histograms and calculated median and standard deviation (SD) of the Relion number of significant samples (*rlnNrOfSignificantSamples*) values in the “run_data.star” file that represent the number of orientations/classes in which each particle participates. Higher numbers mean that the orientation distributions of particles are broader, hence less accurate.

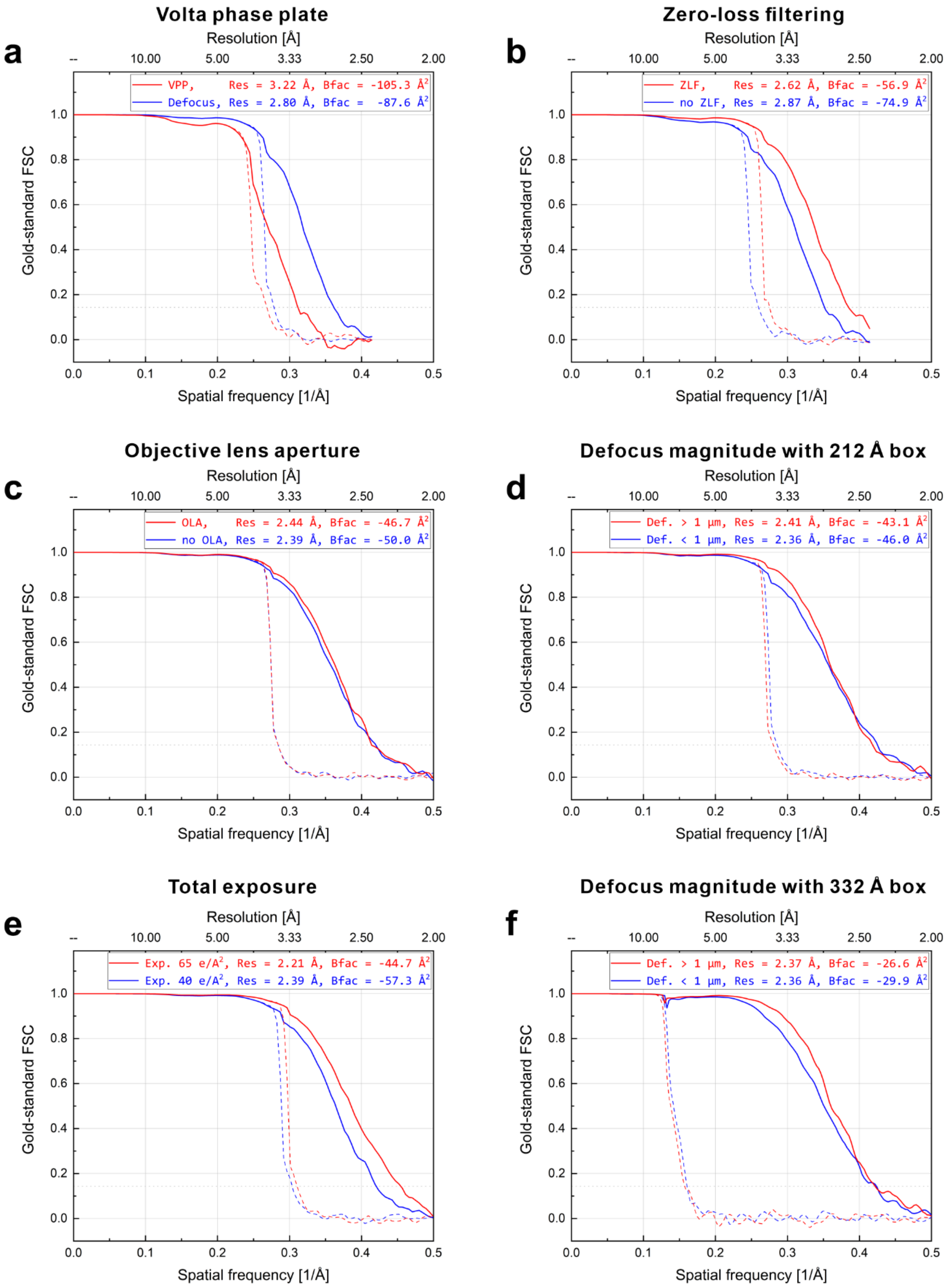

Supplementary Figure 4. Gold-standard Fourier Shell Correlation (FSC) plots for the final 3D refinement of polished particle sets.

a-f Solid lines are the corrected FSCs. Dashed lines are the noise-substituted FSC curves that quantify the effect of the mask and are used to correct the main FSC. The figure legends list the estimated resolution, and the B-factor that was used for sharpening of the map during post-processing. These B-factor values are estimated from the Guinier plot for each map and are not the “true” Rosenthal-Henderson B-factors, as estimated in Supplementary Figure 1.

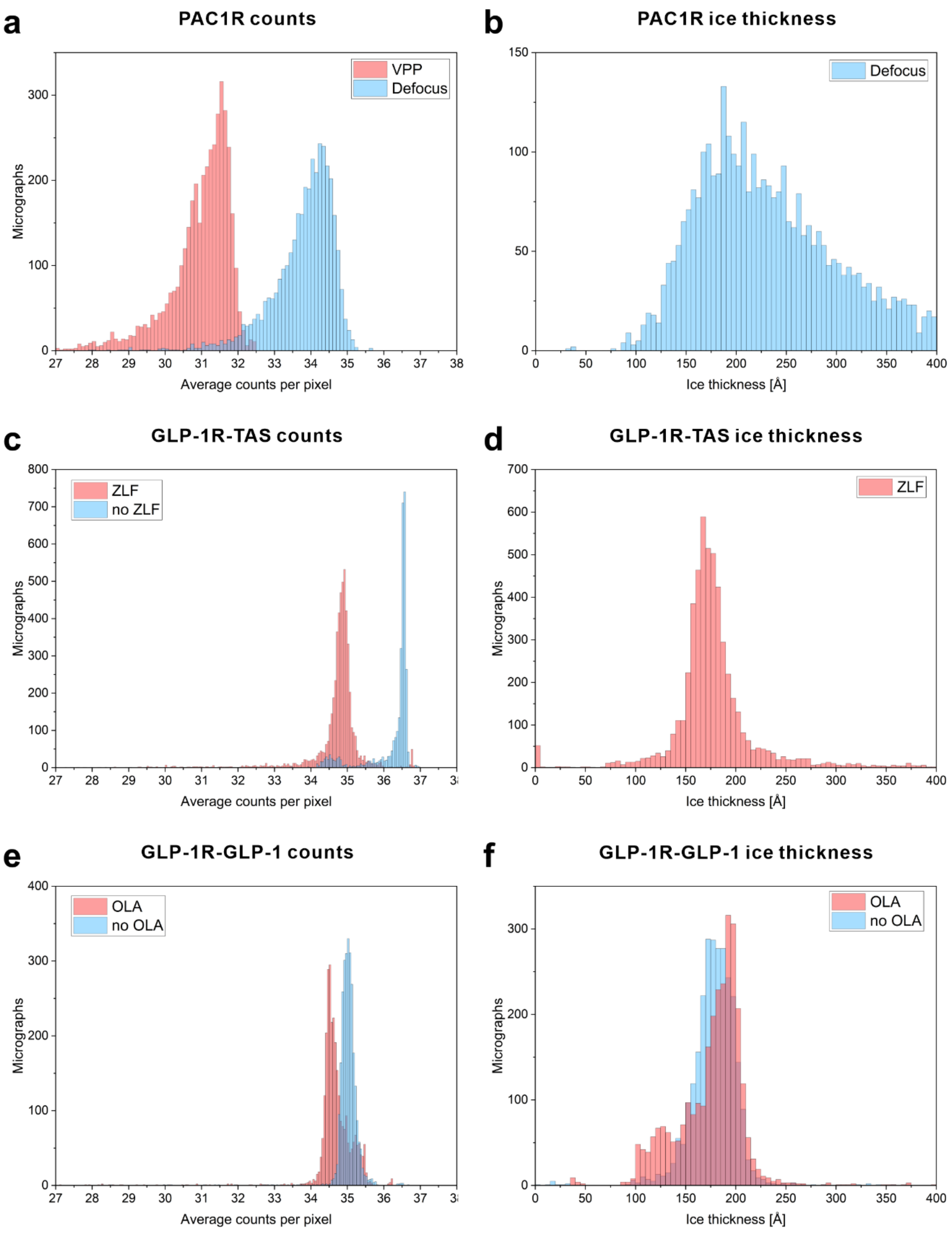

Supplementary Figure 5. Electron event counts and ice thickness statistics for each dataset.
a Average count distribution for all movies in the PAC1R dataset. The VPP reduced the average counts by ~10 % due to electron scattering. **b** Ice thickness distribution of the conventional defocus subset from the PAC1R dataset. The average ice thickness was ~200 Å but there is a long tail towards higher thickness, indicating a variable ice thickness distribution. **c** Average count distribution for the movies in the GLP-1R-TAS dataset. Zero-loss energy filtering reduced the intensity by ~5 % by removing inelastically scattered electrons. **d** Ice thickness distribution of the zero-loss subset of the GLP-1R-TAS dataset. The thickness is in the range of 150 – 200 Å with an average of ~175 Å. **e** Average count distribution of the GLP-1R-GLP-1 dataset. The objective lens aperture intercepted high-angle scattered electrons thus reducing the intensity by 1.4 %. **f** Ice thickness distribution of the GLP-1R-GLP-1 dataset. The thickness was very similar to that of the GLP-1R-TAS dataset in (**d**), with a range of 100 – 220 Å and an average of ~175 Å.

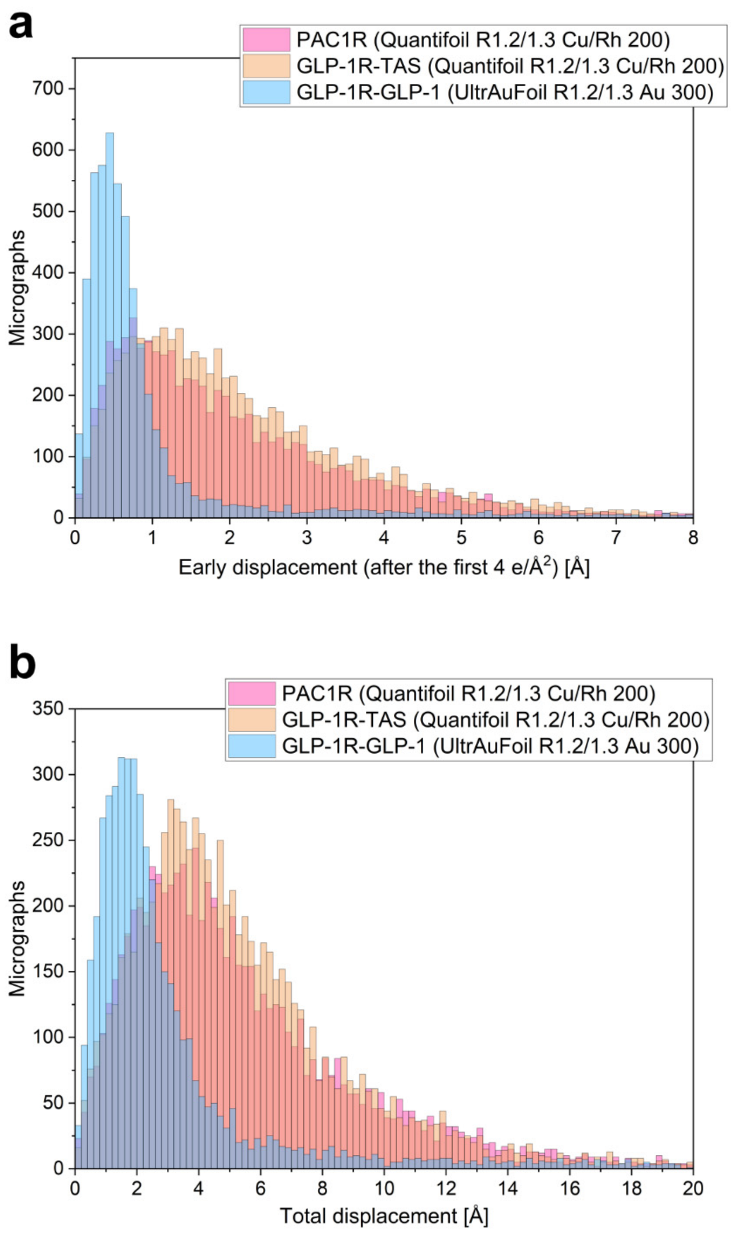

Supplementary Figure 6. Motion statistics from the initial movie motion correction of the datasets.

a Displacement after the first 4 e/Å^2 of the exposure. The GLP-1R-GLP-1 dataset (light blue) collected on a holey gold foil gird exhibited significantly less movement in the beginning of the exposure, which contributed to higher final map resolution (2.1 Å). The PAC1R (pink) and GLP-1R-TAS (beige) had broader initial movement distributions and produced maps at lower resolutions (2.7 and 2.5 Å). **b** The total displacement was also much lower for the GLP-1R-GLP-1 dataset collected on a gold foil support grid.

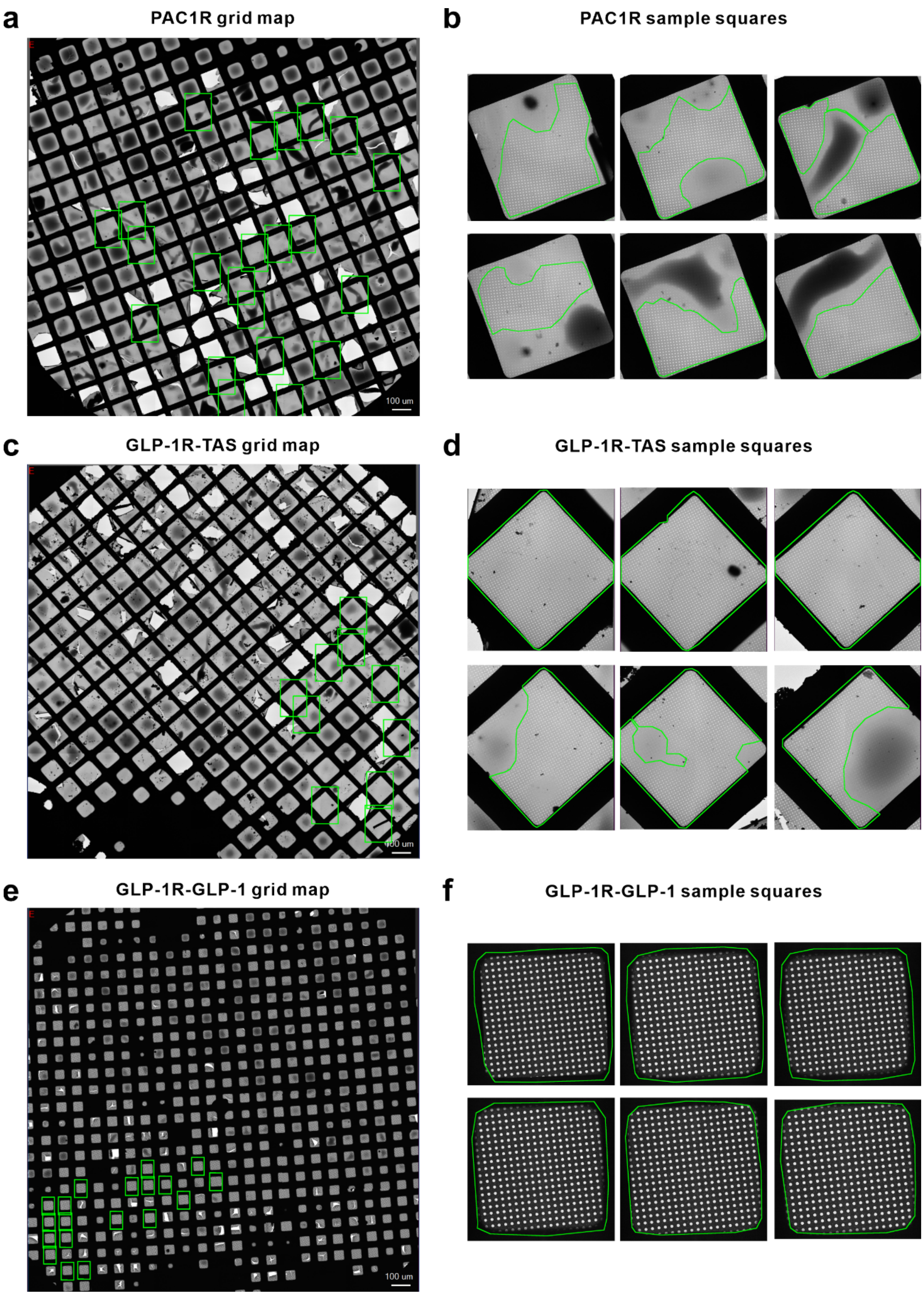

Supplementary Figure 7. Grid maps and representative grids squares from the datasets.

The PAC1R (**a**) and GLP-1R-TAS (**c**) datasets were collected using 200 mesh holey carbon grids. The GLP-1R-GLP-1 (**e**) dataset was collected using a holey gold foil 300 mesh grid. The 200 mesh carbon film grids (**a** and **c**) had more broken squares and more uneven ice distribution. The PAC1R (**a**) grid was glow discharged for 30 s, compared to 90 s for the GLP-1R grids (**c** and **e**). This reduced the drainage of the sample and caused more pooling of sample solution in the middle of squares (**a** and **b** versus **c** and **d**). The gold foil grid (**e** and **f**) had a more uniform ice thickness distribution and less broken squares. Green rectangles in (**a**, **c** and **e**) indicate the squares that were used for data acquisition. Green polygons in (**b**, **d** and **f**) encompass data acquisition areas on the shown squares.

PAC1R: VPP and Defocus subsets processing

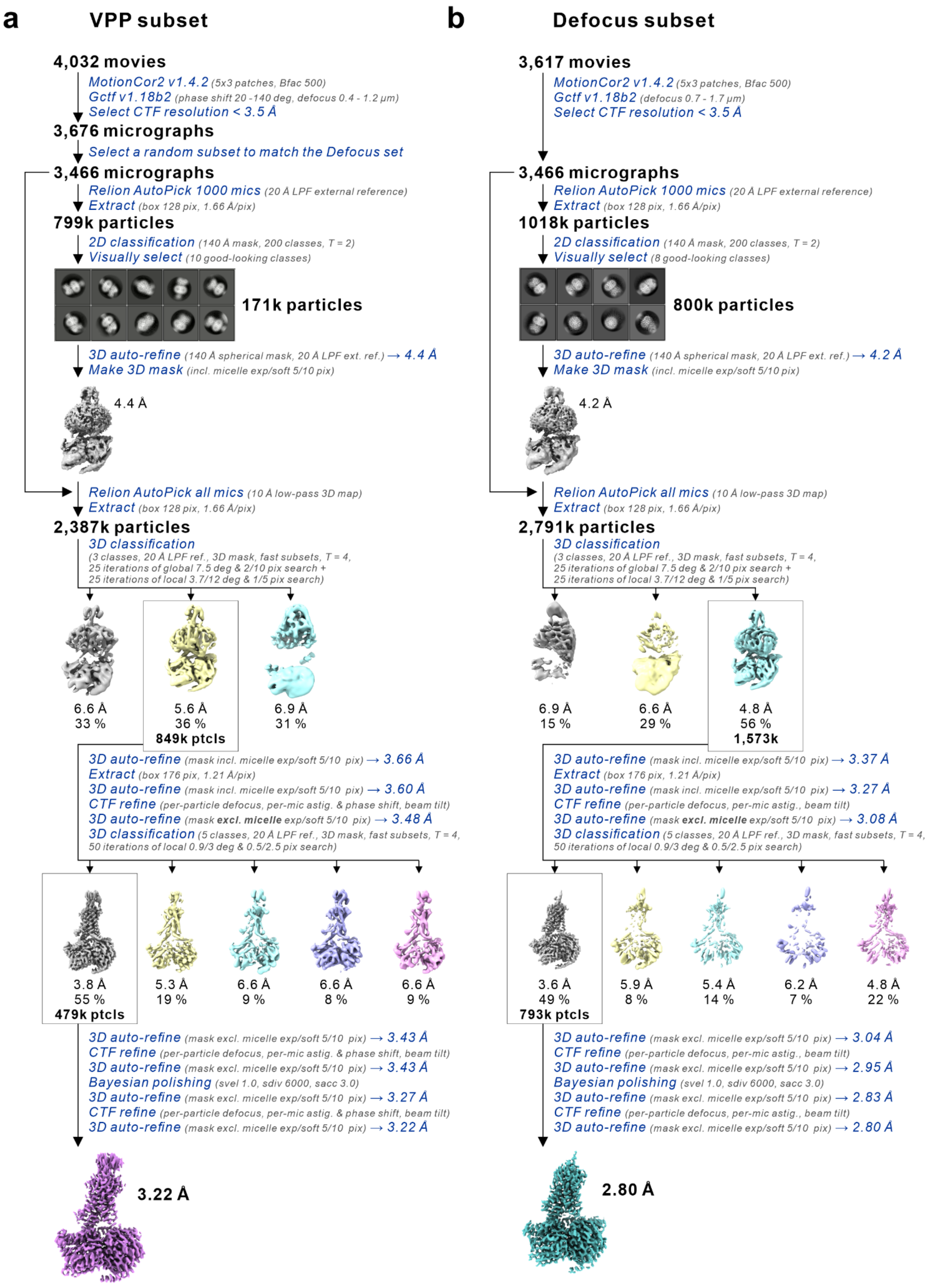

Supplementary Figure 8. PAC1R data processing workflows

a Volta phase plate (VPP) and (**b**) Defocus subsets from the PAC1R dataset.

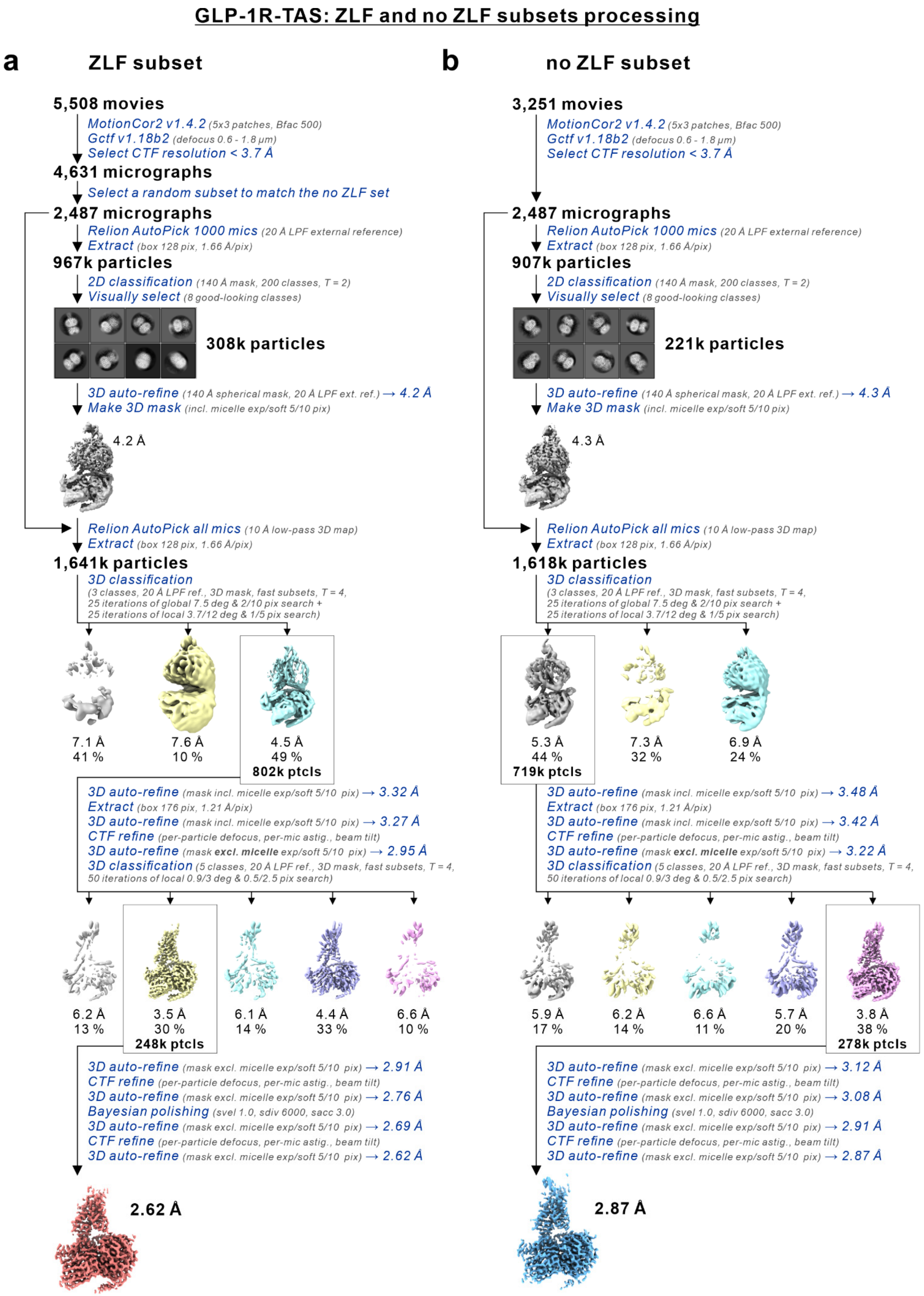

Supplementary Figure 9. GLP-1R-TAS data processing workflows

a Zero-loss energy filtered (ZLF) and (**b**) Non-filtered (no ZLF) subsets from the GLP-1R-TAS dataset.

GLP-1R-GLP-1: OLA and no OLA subsets processing

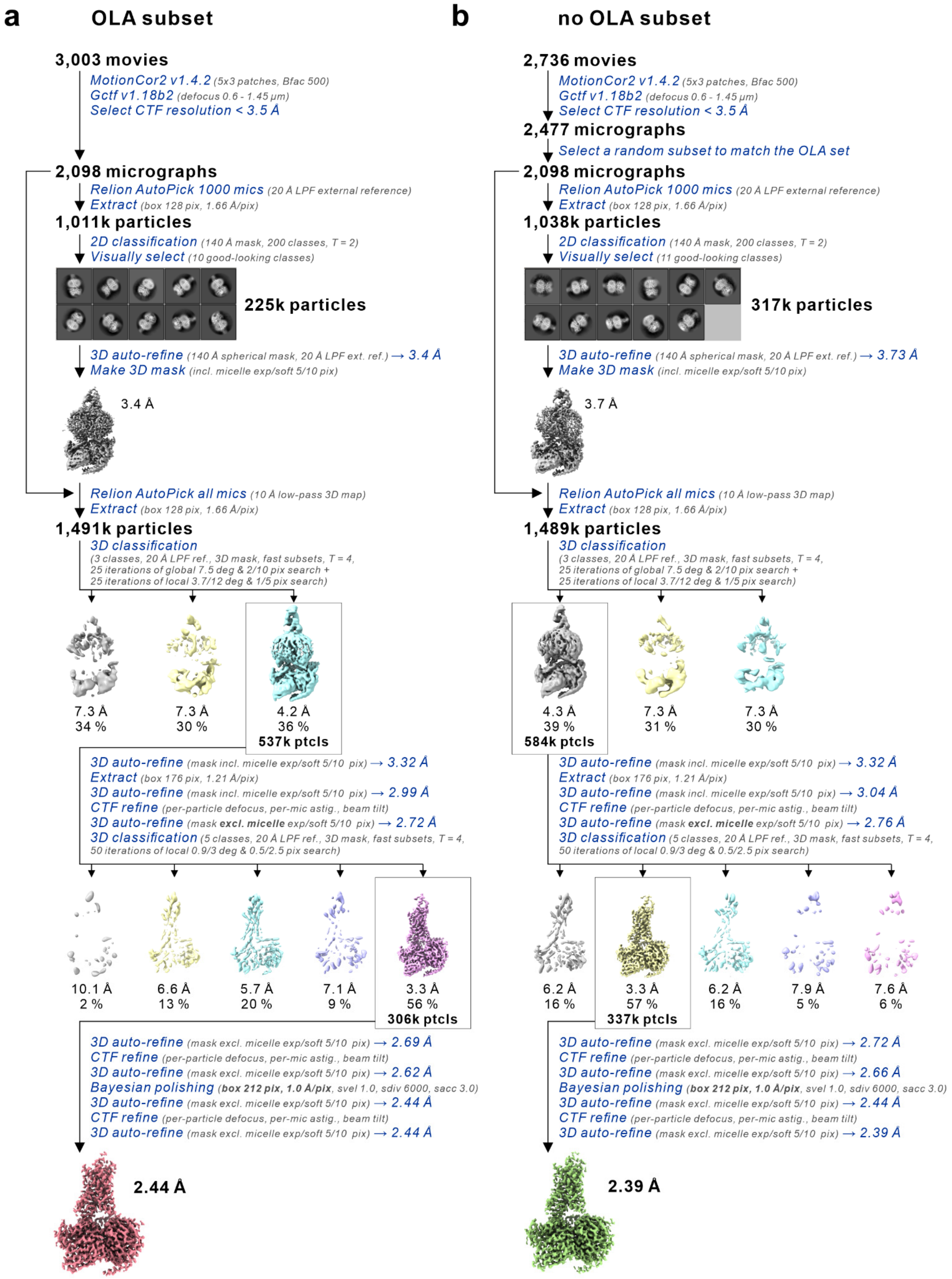

Supplementary Figure 10. GLP-1R-GLP-1 data processing workflows

a Objective lens aperture (OLA) and (**b**) No objective lens aperture (no OLA) subset from the GLP-1R-GLP-1 dataset.

GLP-1R-GLP-1: Def. > 1 µm and Def. < 1 µm subsets processing

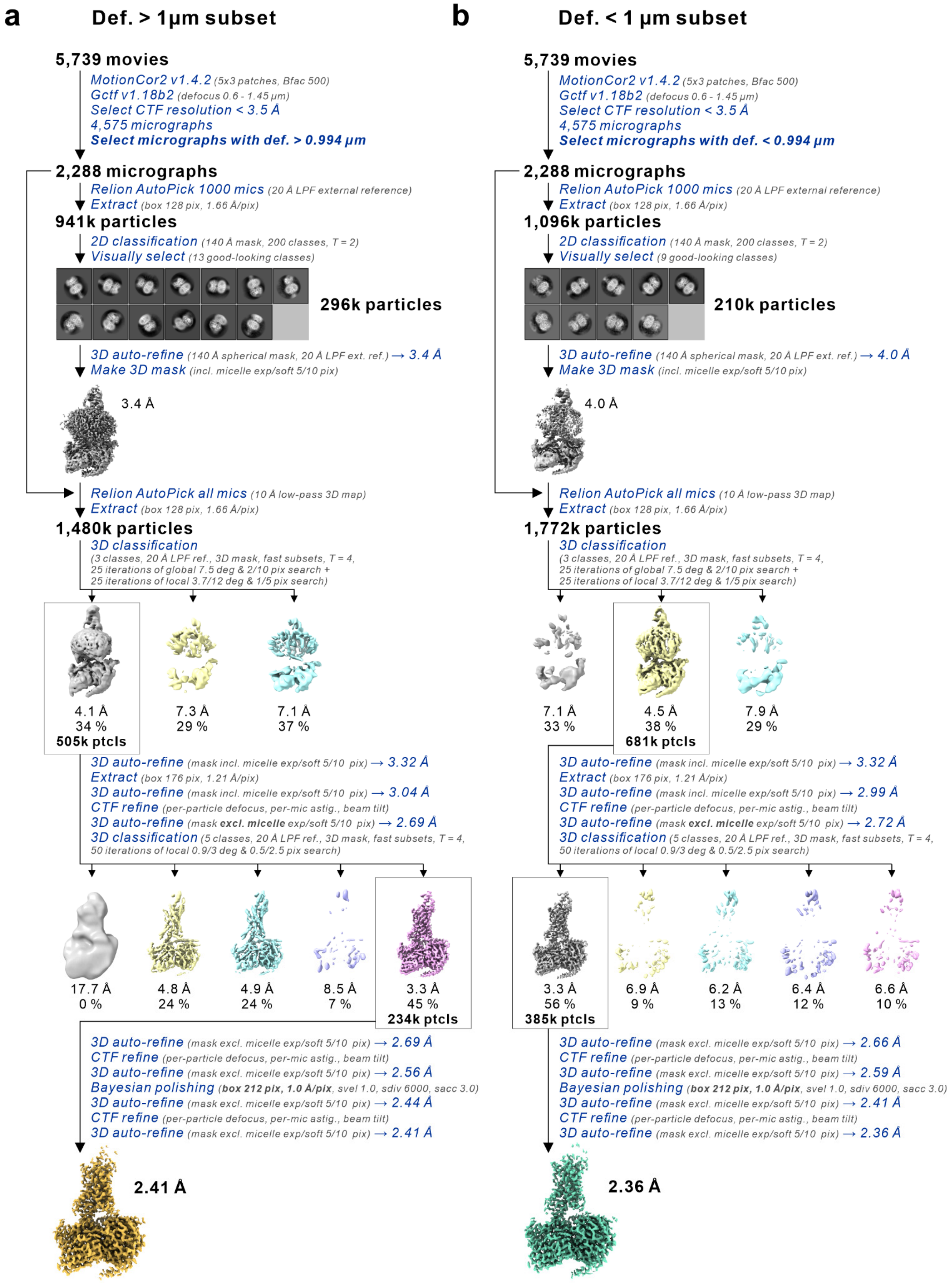

Supplementary Figure 11. GLP-1R-GLP-1 data processing workflows
a High (Def. > 1 µm) and (**b**) Low (Def. < 1 µm) defocus subset from the GLP-1R-GLP-1 dataset.

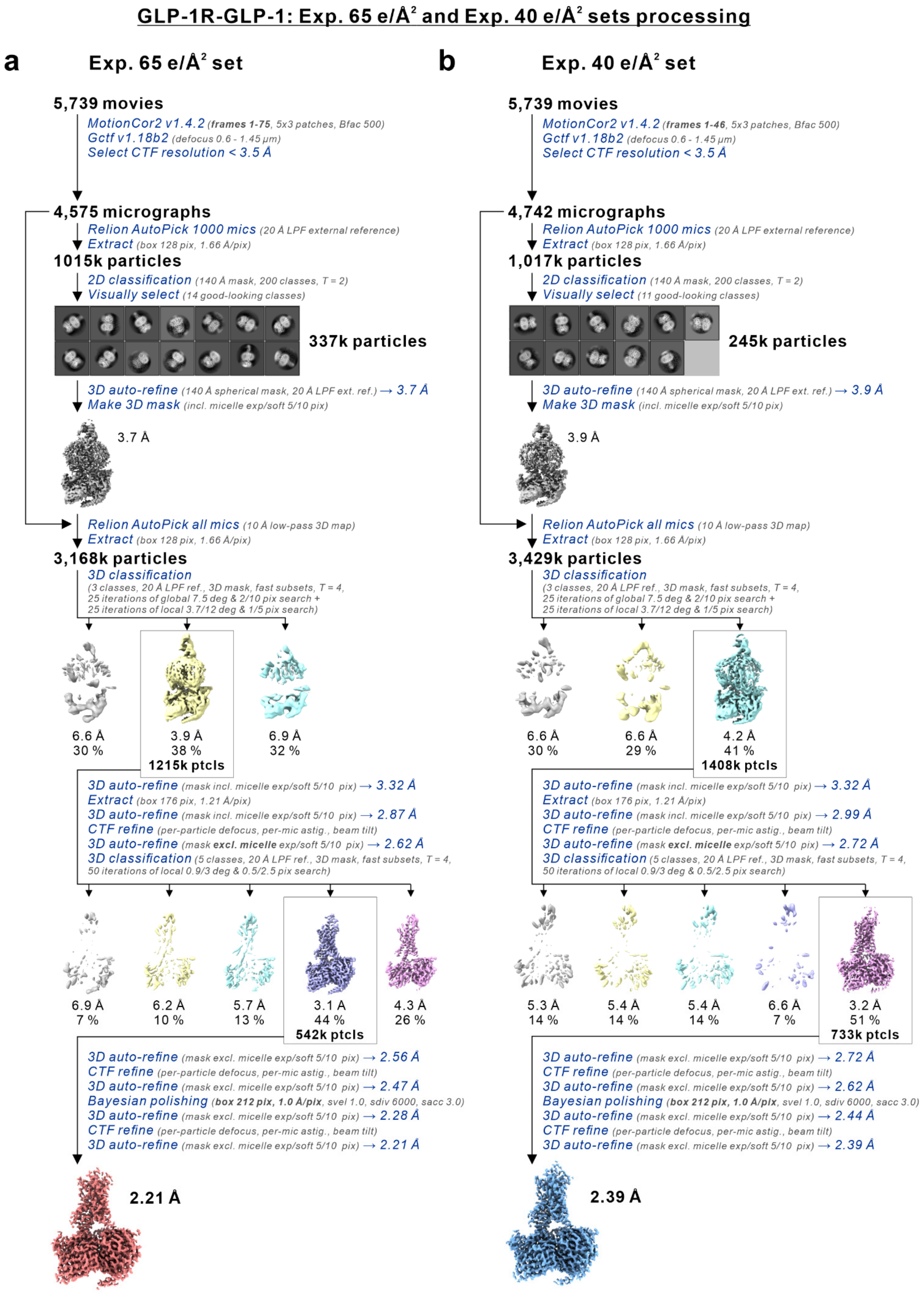

Supplementary Figure 12. GLP-1R-GLP-1 data processing workflows

a Full exposure 65 e/Å^2 and (**b**) Limited exposure 40 e/Å^2 processing of the GLP-1R-GLP-1 dataset.
