# Supplementary material for: Routine sub-2.5 Å cryo-EM structure determination of GPCRs
Source: Nat Commun. 2021 Jul 15;12:4333. doi: 10.1038/s41467-021-24650-3 (PMC8282782; doi:10.1038/s41467-021-24650-3)
Supplement: Supplementary file 3 — Reporting summary [file 41467_2021_24650_MOESM3_ESM.pdf]

nature portfolio

Corresponding author(s): Radostin Danev

Last updated by author(s): Jun 22, 2021

Reporting Summary

Nature Portfolio wishes to improve the reproducibility of the work that we publish. This form provides structure for consistency and transparency in reporting. For further information on Nature Portfolio policies, see our Editorial Policies and the Editorial Policy Checklist.

Statistics

For all statistical analyses, confirm that the following items are present in the figure legend, table legend, main text, or Methods section.

n/a	Confirmed	
☐	☒	The exact sample size (n) for each experimental group/condition, given as a discrete number and unit of measurement
☐	☒	A statement on whether measurements were taken from distinct samples or whether the same sample was measured repeatedly
☒	☐	The statistical test(s) used AND whether they are one- or two-sided<br>*Only common tests should be described solely by name; describe more complex techniques in the Methods section.*
☒	☐	A description of all covariates tested
☒	☐	A description of any assumptions or corrections, such as tests of normality and adjustment for multiple comparisons
☐	☒	A full description of the statistical parameters including central tendency (e.g. means) or other basic estimates (e.g. regression coefficient) AND variation (e.g. standard deviation) or associated estimates of uncertainty (e.g. confidence intervals)
☒	☐	For null hypothesis testing, the test statistic (e.g. F, t, r) with confidence intervals, effect sizes, degrees of freedom and P value noted<br>*Give P values as exact values whenever suitable.*
☒	☐	For Bayesian analysis, information on the choice of priors and Markov chain Monte Carlo settings
☒	☐	For hierarchical and complex designs, identification of the appropriate level for tests and full reporting of outcomes
☒	☐	Estimates of effect sizes (e.g. Cohen's d, Pearson's r), indicating how they were calculated

Our web collection on statistics for biologists contains articles on many of the points above.

Software and code

Policy information about availability of computer code

Data collection	SerialEM v3.7
Data analysis	Relion v3.1.2, MotionCor2 v1.4.2, Gctf v1.18b2

For manuscripts utilizing custom algorithms or software that are central to the research but not yet described in published literature, software must be made available to editors and reviewers. We strongly encourage code deposition in a community repository (e.g. GitHub). See the Nature Portfolio guidelines for submitting code & software for further information.

Data

Policy information about availability of data

All manuscripts must include a data availability statement. This statement should provide the following information, where applicable:

- Accession codes, unique identifiers, or web links for publicly available datasets
- A description of any restrictions on data availability
- For clinical datasets or third party data, please ensure that the statement adheres to our policy

The data needed to evaluate the conclusions of the paper are present in the paper and/or the Supplementary Information. SerialEM scripts and additional data related to this paper may be requested from the authors. The global cryo-EM maps of PAC1R, GLP-1R-TAS, and GLP-1R-GLP-1 are deposited in the Electron Microscopy Data Bank (https://www.ebi.ac.uk/pdbe/emdb/) under accession numbers EMD-0993 [https://www.ebi.ac.uk/pdbe/entry/emdb/EMD-0993], EMD-22883 [https://www.ebi.ac.uk/pdbe/entry/emdb/EMD-22883], and EMD-21992 [https://www.ebi.ac.uk/pdbe/entry/emdb/EMD-21992], respectively. The previously determined atomic models of PAC1R22, GLP-1R-TAS23, and GLP-1R-GLP-124 have been deposited to the Protein Data Bank (https://www.rcsb.org/) under accession codes 6P9Y [https://www.rcsb.org/structure/6P9Y], 7KI1 [https://www.rcsb.org/structure/unreleased/7KI1], and 6X18 [https://www.rcsb.org/structure/6X18], respectively. The complete PAC1R and GLP-1R-GLP-1 datasets have been deposited to the Electron Microscopy Public Image Archive (https://

www.ebi.ac.uk/pdbe/emdb/empiar/) under accession codes EMPIAR-10359 [https://www.ebi.ac.uk/pdbe/emdb/empiar/entry/10359/], and EMPIAR-10673 [https://www.ebi.ac.uk/pdbe/emdb/empiar/entry/10673/], respectively.
We will consider depositing the the GLP-1R-TAS dataset to the Electron Microscopy Public Image Archive, although it is of narrow interest related only to the effect of zero-loss energy filtering. The GLP-1R-GLP-1 dataset, that has already been deposited and is publicly available, is technically more interesting and covers the majority of parameters evaluated in the paper.

Field-specific reporting

Please select the one below that is the best fit for your research. If you are not sure, read the appropriate sections before making your selection.

[x] Life sciences [ ] Behavioural & social sciences [ ] Ecological, evolutionary & environmental sciences

For a reference copy of the document with all sections, see nature.com/documents/nr-reporting-summary-flat.pdf

Life sciences study design

All studies must disclose on these points even when the disclosure is negative.

Sample size	For each GPCR complex, one cryo-EM dataset comprising several thousand micrographs was collected (shown in Table 2). The number of micrographs in each dataset was determined based on our experience with GPCR complexes and the available microscope time.
Data exclusions	As presented in the Methods section (Supplementary Figures 8 - 12) and in accordance with standard cryo-EM practice, micrographs with low estimated CTF resolution were excluded from further processing.
Replication	Each cryo-EM dataset comprises millions of copies of the investigated GPCR complex and therefore has inherent replication. Using random particle subsets during the analyses, as done in this work, is akin to performing multiple experiments.
Randomization	Random particle subsets were used during the 3D auto-refinement steps in Relion and for calculation of Rosenthal-Henderson B-facotr plots.
Blinding	No blinding was necessary because the analyses were based on random particle subsets.

Reporting for specific materials, systems and methods

We require information from authors about some types of materials, experimental systems and methods used in many studies. Here, indicate whether each material, system or method listed is relevant to your study. If you are not sure if a list item applies to your research, read the appropriate section before selecting a response.

Materials & experimental systems

n/a	Involved in the study	
[x]	[ ]	Antibodies
[x]	[ ]	Eukaryotic cell lines
[x]	[ ]	Palaeontology and archaeology
[x]	[ ]	Animals and other organisms
[x]	[ ]	Human research participants
[x]	[ ]	Clinical data
[x]	[ ]	Dual use research of concern

Methods

n/a	Involved in the study	
[x]	[ ]	ChIP-seq
[x]	[ ]	Flow cytometry
[x]	[ ]	MRI-based neuroimaging
